# Living donor liver transplantation for idiopathic portal hypertension with extrahepatic portal vein stenosis and splenic artery aneurysms: a case report and review of the literature

**DOI:** 10.1186/s12893-020-00921-6

**Published:** 2020-10-29

**Authors:** Shigeyuki Kawachi, Naokazu Chiba, Masashi Nakagawa, Toshimichi Kobayashi, Kosuke Hikita, Toru Sano, Koichi Tomita, Hiroshi Hirano, Yuta Abe, Hideaki Obara, Motohide Shimazu

**Affiliations:** 1 Department of Digestive and Transplantation Surgery, Tokyo Medical University Hachioji Medical Center, 1163 Tatemachi, Hachioji, Tokyo 1930998 Japan; 2 Departmet of Diagnostic Pathology, Tokyo Medical University Hachioji Medical Center, 1163 Tatemachi, Hachioji, Tokyo 1930998 Japan; 3 Department of Surgery, Keio University School of Medicine, 35 Shinanomachi, Shinjuku-ku, Tokyo, 1608582 Japan

**Keywords:** Idiopathic portal hypertension, Extrahepatic portal vein stenosis, Living donor liver transplantation, Splenic artery aneurysms, Superficial femoral vein graft

## Abstract

**Background:**

Idiopathic portal hypertension (IPH) generally has a good prognosis and rarely results in liver transplantation. Furthermore, there are few reports of living donor liver transplantation (LDLT) for IPH with extrahepatic portal vein stenosis.

**Case presentation:**

We report the case of a 51-year-old female patient diagnosed with IPH more than 20 years ago. She suffered severe jaundice, massive ascites, and encephalopathy at the time of her visit to our hospital. The patient’s extrahepatic portal vein showed a scar-like stenosis, and the portal flow was completely hepatofugal. Collateral circulation such as the splenorenal shunt was well developed, and multiple splenic artery aneurysms up to 2 cm were observed in the splenic hilum. Her Model for End-Stage Liver Disease score increased to over 40 because of renal dysfunction, requiring temporary dialysis. We performed LDLT using her husband’s right lobe graft and splenectomy. The extrahepatic stenotic portal vein was completely resected, and the superficial femoral vein (SFV) graft collected from the recipient’s right leg was used for portal reconstruction as an interposition graft. Although the clinical course after LDLT had many complications, the patient was discharged on postoperative day 113 and has been fine for 2 years after LDLT. Histopathologically, the explanted liver had obliterative portal venopathy, nodular regenerative hyperplasia, and incomplete septal cirrhosis.

**Conclusion:**

This case showed that severe IPH is occasionally associated with extrahepatic portal vein stenosis and can be treated with LDLT with portal vein reconstruction using an interposition graft. It was also suggested that the SFV is a useful choice for the interposition graft.

## Background

Idiopathic portal hypertension (IPH) comprises disorders that develop increased portal pressure in the absence of cirrhosis [1]. The etiology of IPH is pooly understood as the site of resistance to portal flow is at the presinusoidal level without cirrhosis [2]. Many terms have been used for this disease such as non-cirrhotic portal fibrosis, nodular regenerative hyperplasia (NRH), hepatoportal sclerosis, and idiopathic noncirrhotic portal hypertension; IPH has been the most commonly used term in Japan.

Although most IPH patients have a good prognosis with the treatment for esophageal varices and/or other complications due to portal hypertension, some develop end-stage liver failure and need for liver transplantation [3–5]. It has been reported that about 6% of patients with IPH required liver transplantation [6]. In Japan, there were 8795 living liver transplants until 2017, of which 10 were for IPH patients, including 9 adults and 1 child [7]. Living donor liver transplantation (LDLT) for IPH accounts for about 0.1% of the total, which is an indication of its rarity.

Since IPH is considered to have portal hypertension at the presinusoidal level, there are few reports of LDLT for IPH with extrahepatic portal vein stenosis. Although it is unclear when and how the extrahepatic portal vein stenosis was completed in IPH, this situation required complicated portal vein reconstruction with interposition vein graft, jumping vein graft [8], or renoportal anastomosis [9] using autologous vein graft.

The superficial femoral vein (SFV) seems to be the preferred option for vein grafts because of its caliber, wall thickness, and length obtained. An approximately 15–20 cm segment of the right-side SFV could be usually obtained from the recipient. The collection procedure is technically simple, and postoperative complications such as lower extremity temporary edema are usually not severe, with no permanent complications [10].

Splenic artery aneurysms (SAAs), occurring in 7% to 17% of patients with cirrhosis, often result in catastrophic rupture after liver transplantation [11]. SAAs are closely related to severe portal hypertension with large portosystemic collaterals. While visceral aneurysms larger than 2 cm are more likely to rupture, preventive treatment should be encouraged regardless of the size of SAAs, because the risk of rupture increases during the perioperative period of liver transplantation, in which hemodynamic and proteolytic changes occur [11, 12]. Whether to select interventional radiology (IVR) or surgical treatment such as splenectomy as a treatment method needs to be individually selected according to the patient’s general condition, and the location and number of SAAs.

We report a very rare case of end-stage IPH with extrahepatic portal vein stenosis and SAAs close to the hilum of the spleen. As the patient was so sick that she had to temporarily undergo dialysis, she could not get any preventive treatment against the SAAs before liver transplantation. We decided to perform LDLT and splenectomy with a complicated portal vein reconstruction using the SFV graft.

## Case presentation

**Fig. 1:**
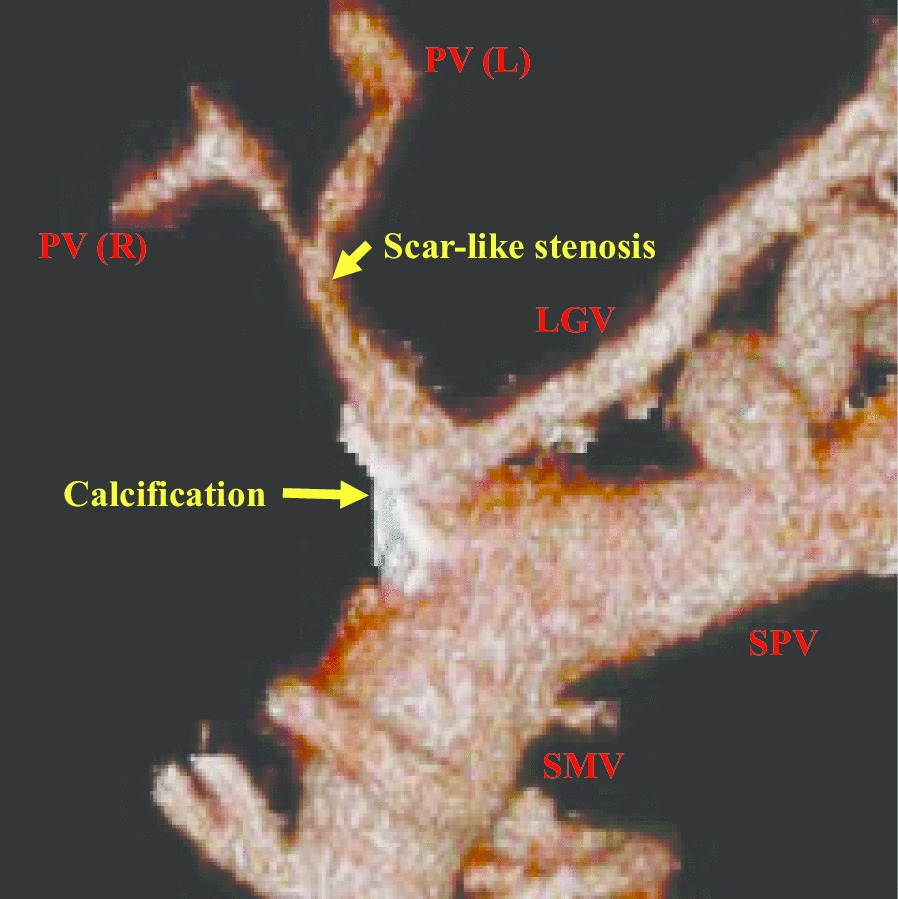
A 3D preoperative portal venous image. The extrahepatic portal vein showed a scar-like stenosis, and collateral circulation was well developed. Dense calcification was observed at the site of the splenomesenteric junction

**Fig. 2:**
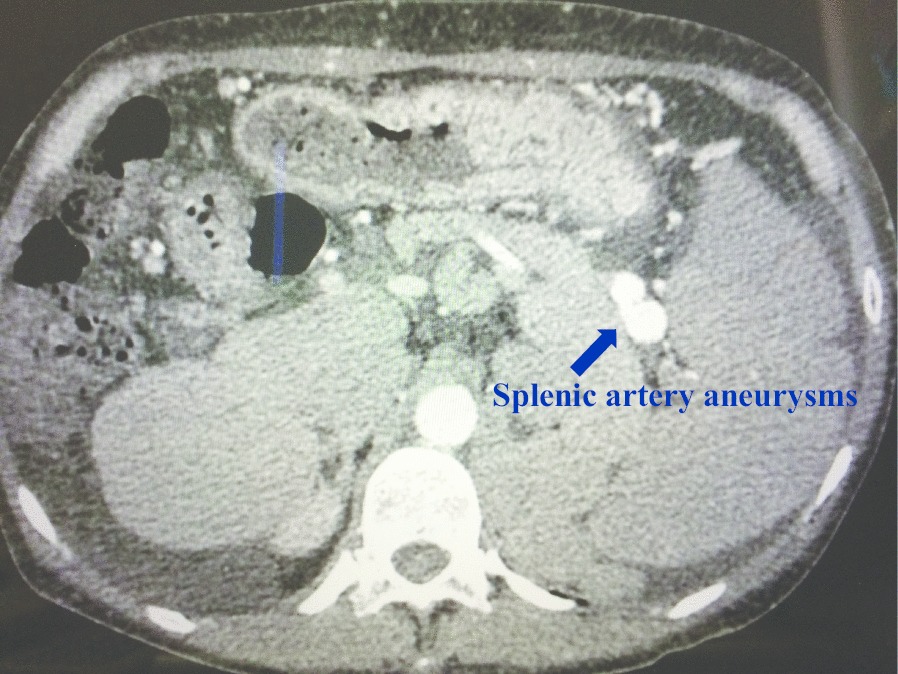
The arterial phase of abdominal dynamic CT. The arterial phase of abdominal dynamic CT reveals the multiple splenic artery aneurysms up to 2 cm in size in the splenic hilum

A 51-year-old female patient was admitted to explore the possibility of liver transplantation for liver failure due to IPH. She had NRH on liver biopsy 20 years ago and was diagnosed with IPH. The patient’s extrahepatic portal vein showed a scar-like stenosis, and the portal flow was completely hepatofugal. Collateral circulation such as the splenorenal shunt was well developed, and dense calcification was observed at the site of splenomesenteric junction (Fig. 1), suggesting that the usual portal reconstruction would be impossible. Furthermore, multiple splenic artery aneurysms (SAAs) up to 2 cm were observed in the splenic hilum (Fig. 2). The patient’s condition was getting worse and her Child–Pugh score became 13, and her Model for End-Stage Liver Disease (MELD) score increased to over 40 because of renal dysfunction, requiring temporary dialysis. Due to the high risk of rupture during the perioperative period of liver transplantation, it was considered necessary to take some preventive measures against the SAAs; however, it was difficult to perform preoperatively due to her medical condition. Therefore, splenectomy at the time of transplantation was needed. Since the splenorenal shunt was likely to be destroyed during splenectomy, portal vein reconstruction using renoportal anastomosis was considered to not be possible. For the jumping vein graft, long-term patency was a concern because it passed through a non-physiological route. We decided to use an interposition autologous graft between the splenomesenteric junction and the graft portal vein stump.

**Fig. 3:**
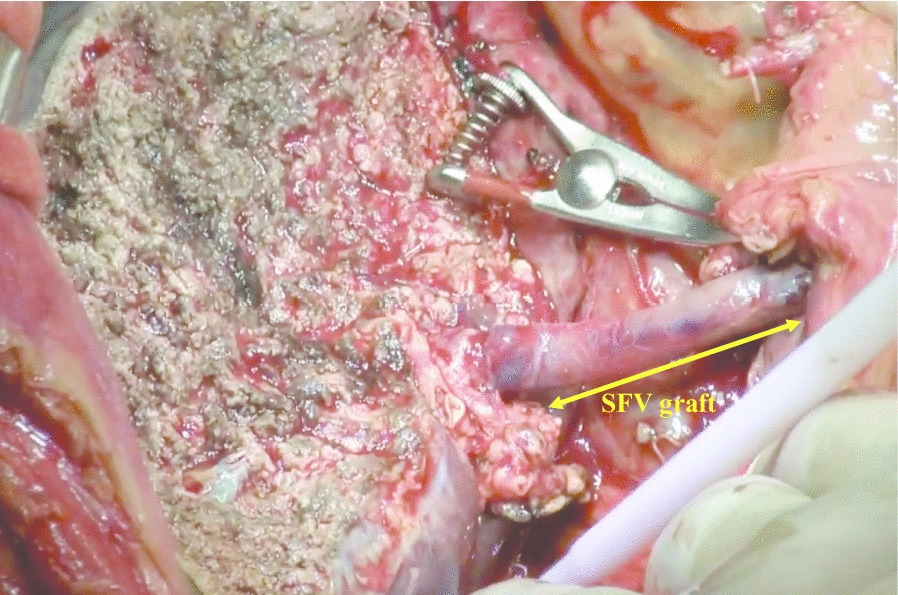
Intraoperative photo after portal vein reconstruction. Portal reconstruction was performed using the interposition SFV graft between the right lobe graft portal vein stump and the splenomesenteric junction

LDLT using her husband’s right lobe graft was performed. The graft recipient weight ratio (GRWR) of the graft was 1.26, which was rated as a sufficient large for the recipient. After total hepatectomy, the recipient’s right side SFV graft was harvested. The portal vein was dissected to the side of the pancreas, and the dorsal side of the pancreas was tunneled to confirm the splenic vein (SPV) and superior mesenteric vein (SMV). After clamping the SMV and SPV, the stenotic portal vein was completely resected, and the dense calcification of vascular endothelium was removed at the splenomesenteric junction. The SFV graft was anastomosed to the splenomesenteric junction in an end-to-side fashion. After right hepatic vein reconstruction and confirming the sufficient front flow of the portal vein, an end-to-end anastomosis was performed on the portal vein of the graft liver and the SFV graft (Fig. 3).

**Fig. 4:**
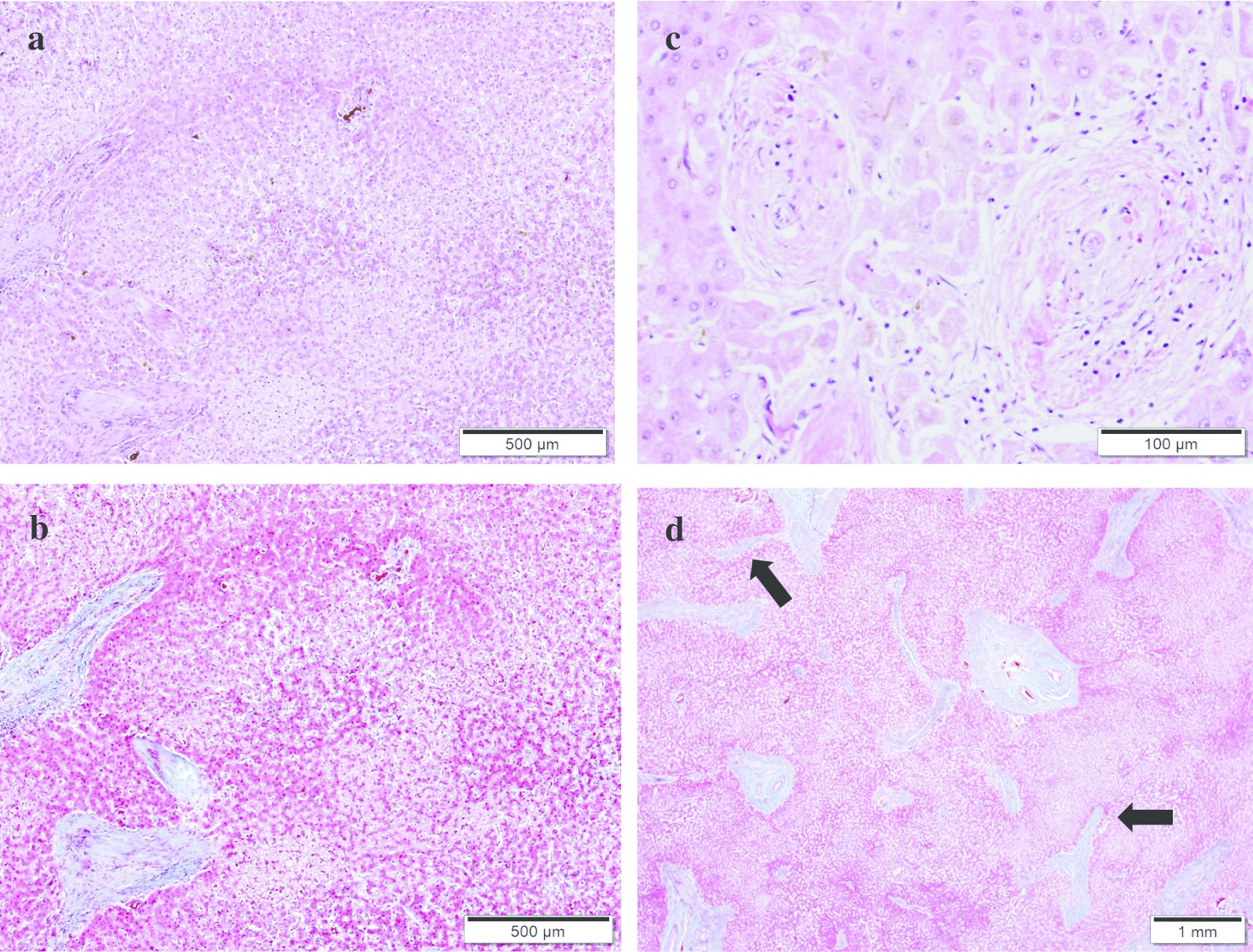
Histologic feature of the explanted liver. **a** Nodular regenerative hyperplasia (NRH) was noted in the liver explants. Parenchymal nodularity created by areas of hypertrophic hepatocytes in periportal areas alternating with areas of cord atrophy without fibrosis (H&E stain). **b** Same field of view as A by Masson trichrome stain. **c** Obliterative portal venopathy was seen in this portal tract, in which there is a diminished-caliber and almost sclerotic portal vein (H&E stain). **d** Incomplete septal cirrhosis was observed in this slide. Here, fibrous bands (arrows) extend from the portal tract and end blindly in the liver parenchyma (Masson trichrome stain)

The clinical course after transplantation had many complications. An intraabdominal abscess due to grade B pancreatic fistula classified by international study group of pancreatic fistula (ISGPF) [13] after splenectomy for splenic artery aneurysm occurred on postoperative day (POD) 14. It was gradually relieved by percutaneous CT guide drainage. Late onset bile leakage occurred on POD 32, which was managed by drainage and biliary stent. A large amount of ascites appeared and the anastomotic stenosis between the SFV graft and the splenomesenteric junction was found on POD 52. A small laparotomy was performed to secure the ileocolic vein, and balloon dilatation (diameter of 8 mm) of anastomotic stenosis was achieved by interventional radiology technique via the ileocolic venous approach. The pressure difference before and after the anastomosis site was 15 mmHg before balloon dilatation, and 3 mmHg after the treatment. Massive ascites had been gradually improved and the patient was discharged on POD 113. The explant liver histologically showed obstructive portal venopathy, NRH, and incomplete septal cirrhosis, which was typical of IPH (Fig. 4).

**Fig. 5:**
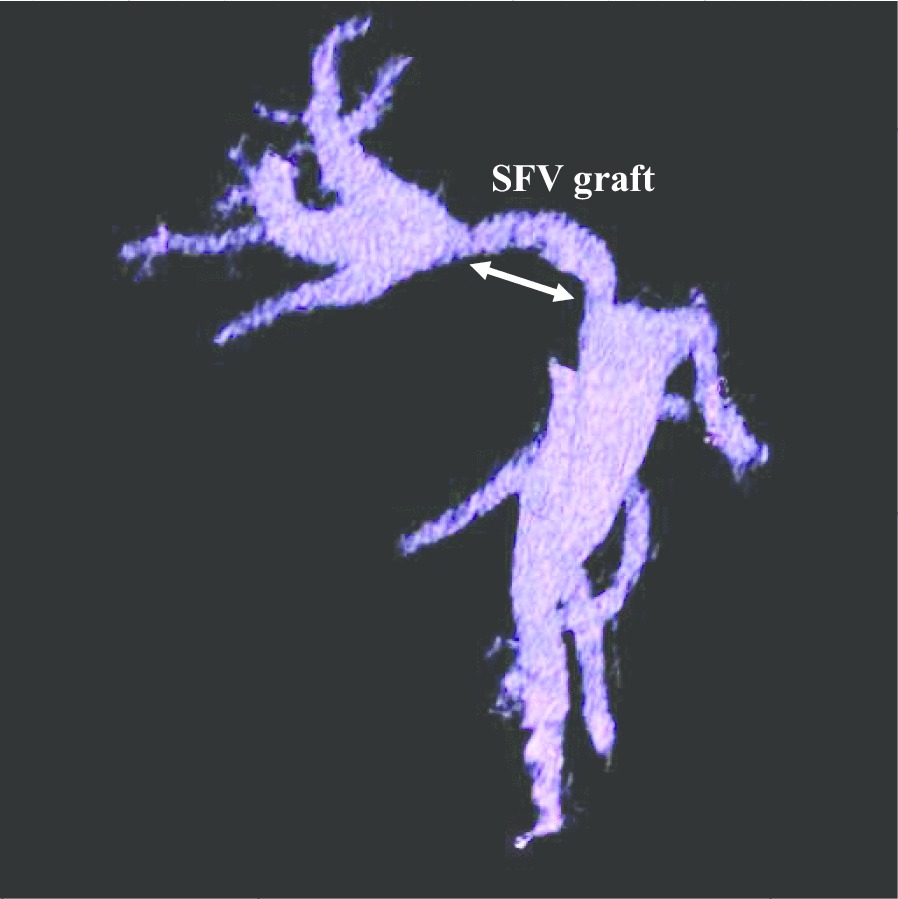
Portal venous image 2 years after LDLT. Portal venous image was constructed two years after LDLT. The interposition SFV graft was well patent and no anastomotic stenosis was observed in both, the distal and proximal anastomotic sites

The clinical course after discharge was uneventful and the SFV graft patency was excellent at 2 years after LDLT (Fig. 5).

## Discussion and conclusion

IPH is a rare disease characterized by portal hypertension without cirrhosis. The prognosis of IPH is considered as feasible with management for esophageal varices and/or splenomegaly by supportive treatments such as endoscopic, radiological, and surgical approach. The definite etiology of IPH is still uncertain, however, there are several theories that have been attributed to the potential pathogenesis of IPH. The contributing factors include immunological disorders, infections, and genetic variations [1].

IPH rarely results in neither end-stage liver failure nor requiring liver transplantation. Past case reports for severe IPH requiring liver transplantation were summarized in Table 1 [3, 5, 9, 14–20]. To date, about 50 cases of liver transplantation have been reported for IPH. Approximately 80% of the reported cases were male, and few patients had been diagnosed with IPH preoperatively. Most of cases were cadaveric liver transplantation cases for cryptogenic cirrhosis, and reports of LDLT cases were even rarer, and were limited to reports from India and Japan. Out of 8795 LDLT in Japan up to 2017, only 10 (0.1%) cases for IPH. The same pathological condition as IPH is called for Non-cirrhotic portal fibrosis (NCPF) in India. Saigal et al. reported that 10 (2.5%) out of 406 LDLT cases were pathologically diagnosed as pure NCPF from the study of explant livers in India. This rate was extremely high as compared to that in Japan, however, whether or not the IPH in Japan was the same condition as NCPF in India may need further verification.

**Table 1:** Summary of reports of IPH requiring liver transplantation

Authors	Year	Patients no	Gender	Diagnosis before LT	LT	Pathological diagnosis	Prognosis
McDonald et al. [14]	1990	1	Male	Cirrhosis	OLT	NRH	Died (4 months)
Elariny et al. [15]	1994	1	Female	Cirrhosis	OLT	NRH	Alive
Bernard et al. [16]	1995	1	Male	Cirrhosis	OLT	ISC	Alive
Loinaz et al. [17]	1998	4	Male 4	NRH 1, cirrhosis 1, nodular liver 1, chronic liver disease 1	OLT	NRH 3, partial nodular transformation 1	Alive 2, died 2 (1 month, 3 months)
Radomski et al. [18]	2000	4	Male 3 Female 1	Cirrhosis 4	OLT	NRH 4	Alive 4
Dumortier et al. [19]	2001	8	Male 8	IPH 8	OLT	NRH 3, ISC 5	Alive 8
Krasinskas et al. [3]	2005	16	Male 11 Female 5	Cirrhosis 13, NCPH related 3	OLT	Portal vascular abnormalities 16, NRH 15, ISC 9	Alive 14, died 1 (5 months), retransplantation 1
Inokuma et al. [5]	2009	1	Female	IPH	LDLT	Portal vascular abnormalities	Died (5 months)
Saigai et al. [20]	2011	10	Male 8 Female 2	Cryptogenic cirrhosis 9, alcoholic cirrhosis 1	LDLT	Pure NCPF	Unknown 10
Matsumoto et al. [9]	2013	1	Male	Cryptogenic cirrhosis, atrophied portal venous trunk	LDLT, renoportal anastomosis	IPH	Alive
Our case	2020	1	Female	IPH, extrahepatic portal vein stenosis	LDLT, interposition SFV graft	IPH	Alive
Total		48	Male 38 Female 10	Extrahepatic portal vein abnormalities 2	OLT 35 LDLT 13		Died 5, alive 33, unknown 10

*LT* liver transplantation, *OLT* orthotopic liver transplantation, *LDLT* living donor liver transplantation, *NRH* nodular regenerative hyperplasia, *ISC* incomplete septal cirrhosis, *IPH* idiopathic portal hypertension

When the disease duration of IPH had been long, the extrahepatic portal vein itself may occasionally become atrophic and scar-like because of the hepatofugal portal flow and/or unknown reasons. These situations may result in stenosis of the extrahepatic portal vein. To date, there is only one case report of liver transplantation for IPH with extrahepatic portal vein stenosis. In that case, a right lobe LDLT with renoportal anastomosis was performed using the recipient’s internal jugular vein graft for portal reconstruction [9]. However, in our case, splenectomy for splenic artery aneurysms might destroy a part of the splenorenal shunt, suggesting that renoportal anastomosis might be impossible. Alternatively, portal vein reconstruction with interposition vein graft between the splenomesenteric junction and donor portal vein stump was performed using a recipient’s superficial femoral vein (SFV).

The SFV graft obtained from the recipient is not only an autologous vein graft with no immune reaction, but also a very long vein graft with a sufficient diameter. A 15–20 cm length graft can be usually obtained, so the SFV graft alone can cover all the vein graft reconstruction in LDLT [8, 21]. The procurement procedure is simple, but the length of the skin incision may be longer than that of other vein grafts. Since SFV grafts have valves in their lumens, we have dissected all these valves under the direct vision. It is well known that harvesting the SFV peripherally to the profound femoral vein inflow is well tolerated and associated with only minimal lower extremity venous morbidity [10]. The lower extremity suffered edema, but it improved within about 1 month, and did not pose long-term problem.

In our case, anastomotic stenosis between the splenomesenteric junction and SFV graft occurred on POD 52, resulting in increasing ascites. Conservative treatments such as the use of diuretics did not succeed; the re-operation and balloon dilatation of the anastomotic stenosis were performed using the interventional radiology technique via the ileocolic vein. Possible reasons of anastomotic stenosis might include technical errors, and/or a twisting associated with the graft regeneration, but it is not clear. Despite the reason being unclear, a single attempt of balloon dilatation had completely solved the problem, therefore, we determined that this complication should not raise doubts about the usefulness of the SFV graft in portal reconstruction.

The diagnosis of IPH is extremely difficult because the liver is usually atrophic and closely resembles cirrhosis in both clinical symptoms and diagnositic imaging in the late stage of IPH. Therefore, only histological findings obtained by liver biopsy will lead to the precise diagnosis of IPH preoperatively. Histopathologically, almost all patients with IPH have some degree of obliterative portal venopaty, furthermore, NRH and incomplete septal cirrhosis are also characteristic findings of IPH. Our patient had an NRH on liver biopsy and was diagnosed with IPH 20 years before the first visit to our hospital. The patient’s explanted liver showed evidence of obliterative portal venopathy, NRH, and incomplete septal cirrhosis, which were compatible with IPH, but not with end-stage cirrhosis.

In conclusion, we reported a very rare case of end-stage IPH with extrahepatic portal vein stenosis and the SAAs close to the hilum of the spleen. This case showed that severe IPH is occasionally associated with extrahepatic portal vein stenosis and can be treated with LDLT with portal vein reconstruction using an interposition graft. It was also suggested that the SFV is a useful choice for the interposition graft.

## Abbreviations

IPH: Idiopathic portal hypertension
NRH: Nodular regenerative hyperplasia
LDLT: Living donor liver transplantation
SFV: Superficial femoral vein
SAA: Splenic artery aneurysm
SPV: Splenic vein
SMV: Superior mesenteric vein
POD: Postoperative day
NCPF: Non-cirrhotic portal fibrosis

## Availability of data and materials

The clinical datasets supporting the conclusions of this study were derived from the patient electronic files. Therefore, restrictions to availability apply due to data protection regulations. Anonymized data are, however, available from the corresponding author on reasonable request and with permission of the Tokyo Medical University Hachioji Medical Center and the local review board.
